# Effectiveness of polymyxin b immobilized fiber hemoperfusion in patients with septic shock due to Gram-negative bacillus infection: the PMXHP study

**DOI:** 10.1186/cc14211

**Published:** 2015-03-16

**Authors:** N Saito, K Sugiyama, T Ohnuma, T Kanemura, M Nasu, Y Yoshidomi, H Adachi, H Koami, Y Tsujimoto, A Tochiki, Y Wagatsuma, T Myumi

**Affiliations:** 1 Chiba Hokusou Hospital, Nippon Medical School, Chiba, Japan; 2 Tokyo Metropolitan Bokutoh Hospital, Tokyo, Japan; 3 Saitma Medical Center, Jichi Medical University, Saitama, Japan; 4 National Hospital Organization Disaster Medical Center, Tokyo, Japan; 5 Urasoe General Hospita, Okinawa, Japan; 6 Saga-ken Medical Center, Koseikan, Saga, Japan; 7 Iizuka Hospital, Fukuoka, Japan; 8 Saga University Hospital, Saga, Japan; 9 Yamagata Prefectural Central Hospital, Yamagata, Japan; 10 Tsukuba Medical Center Hospital, Ibaraki, Japan; 11 University of Tsukuba, Ibaraki, Japan; 12 University of Occupational and Environment Health, Fukuoka, Japan

## Introduction

Mortality from septic shock in the ICU remains high, ranging from 30 to 50%. In particular, Gram-negative bacilli (GNB) account for 40% of the causative bacteria of severe sepsis, which progresses to multiorgan failure due to significant inflammation. Hemoperfusion with polymyxin B-immobilized fiber (PMX) adsorbs endotoxin and can reduce the inflammatory cascade of sepsis due to GNB. However, the clinical efficacy of this treatment has not been demonstrated. We aimed to verify the efficacy of endotoxin adsorption therapy by using PMX.

## Methods

We retrospectively evaluated 387 patients who received a broad-spectrum antimicrobial treatment for septic shock due to GNB between January 2009 and December 2012 in the ICU of 10 Japanese tertiary hospitals. After alignment of the treatment time phase for each patient, we divided the patients into two groups according to whether PMX treatment was performed within 24 hours after ICU admission (PMX group: *n *= 129 and non-PMX group: *n *= 258). The primary endpoint was 28-day mortality.

## Results

The mean (SD) age and SOFA scores on ICU admission were 72.5 (12.5) years and 10.0 (3.4), respectively. The infection site was intra-abdominal (47.0%), pulmonary (17.6%), and urinary tract (27.8%). Two-thirds of all patients had bacteremia due to GNB. No difference in 28-day mortality was observed between the two groups (PMX: 33.9% vs. non-PMX: 33.1%, *P *= 0.87). In the Cox regression analysis adjusted for age, sex and facilities, the PMX treatment (hazard ratio = 0.87; 95% confidence interval, 0.53 to 1.43) did not improve the outcome.

## Conclusion

No difference in mortality rate was observed after adjustment for the endotoxin adsorption therapy with PMX in the patients with septic shock due to GNB.

